# Host traits and temperature predict biogeographical variation in seagrass disease prevalence

**DOI:** 10.1098/rspb.2024.3055

**Published:** 2025-02-12

**Authors:** F. R. Schenck, J. K. Baum, K. E. Boyer, J. E. Duffy, F. J. Fodrie, J. Gaeckle, T. C. Hanley, C. M. Hereu, K. A. Hovel, P. Jorgensen, D. L. Martin, N. E. O’Connor, B. J. Peterson, J. J. Stachowicz, A. R. Hughes

**Affiliations:** ^1^ Massachusetts Division of Marine Fisheries, 30 Emerson Avenue, Gloucester, MA, USA; ^2^ Department of Biology, University of Victoria, PO Box 1700 STN CSC, Victoria, British Columbia, Canada; ^3^ Estuary and Ocean Science Center, San Franscisco State University, 3150 Paradise Drive, Tiburon, CA, USA; ^4^ MarineGEO Program, Smithsonian Environmental Research Center, 647 Contees Wharf Road, Edgewater, MD, USA; ^5^ Institute of Marine Science, University of North Carolina at Chapel Hill, 3431 Arendell Street, Morehead City, NC, USA; ^6^ Nearshore Habitat Program, Washington State Department of Natural Resources, Aquatic Resources Division, 1111 Washington Street SE, Olympia, WA, USA; ^7^ Department of Biology, Sacred Heart University, 5151 Park Avenue, Fairfield, CT, USA; ^8^ Facultad de Ciencias Marinas, Universidad Autonoma de Baja California, Carretera Tijuana-Ensenada 3917, Ensenada, Baja California, Mexico; ^9^ Department of Biology, Coastal and Marine Institute, San Diego State University, 550024 Campanile Drive, San Diego, CA, USA; ^10^ Instituto de Investigaciones Marinas y Costeras (IIMyC-UNMdP-CONICET), Juan B. Justo 2550, Mar del Plata, Buenos Aires, Argentina; ^11^ Department of Biology, University of North Florida, 1 UNF Drive, Jacksonville, FL, USA; ^12^ Department of Zoology, School of Natural Sciences, Trinity College Dublin, College Green, Dublin, Ireland; ^13^ School of Marine and Atmospheric Sciences, Stony Brook University, 239 Montauk Highway, Southampton, NY, USA; ^14^ Department of Evolution and Ecology, University of California Davis, 1 Shields Avenue, Davis, CA, USA; ^15^ Coastal Sustainability Institute, Northeastern University, 430 Nahant Road, Nahant, MA, USA

**Keywords:** biogeography, eelgrass, disease triangle, host–parasite interactions, *Labyrinthula zosterae*, wasting disease, *Zostera marina*

## Abstract

Diseases are ubiquitous in natural systems, with broad effects across populations, communities and ecosystems. However, the drivers of many diseases remain poorly understood, particularly in marine environments, inhibiting effective conservation and management measures. We examined biogeographical patterns of infection in the foundational seagrass *Zostera marina* by the parasitic protist *Labyrinthula zosterae*, the causative agent of seagrass wasting disease, across >20° of latitude in two ocean basins. We then identified and characterized relationships among wasting disease prevalence and a suite of host traits and environmental variables. Host characteristics and transmission dynamics explained most of the variance in prevalence across our survey, yet the particular host traits underlying these relationships varied between oceans, with host size and nitrogen content important in the Pacific and host size and density most important in the Atlantic. Temperature was also a key predictor of prevalence, particularly in the Pacific Ocean. The strength and shape of the relationships between prevalence and some predictors differed in our large-scale survey versus previous experimental and site-specific work. These results show that both host characteristics and environment influence host–parasite interactions, and that some such effects scale up predictably, whereas others appear to depend on regional or local context.

## 1. Introduction

Infectious disease is a major determinant of animal and plant population dynamics [1]. Disease prevalence varies in space owing to a combination of host traits and biotic and abiotic environmental conditions, in addition to characteristics of the parasites themselves [2–4]. For instance, soil moisture, soil biota and host habitat type all significantly influenced infection outcomes in wild flax (*Linum marginale*) host populations [5]. Similarly, host density, host genetic identity and multiple abiotic environmental factors (e.g. temperature and salinity) affect the prevalence, intensity and virulence of a protozoan-induced disease in the eastern oyster (*Crassostrea virginica*) [6–9]. Identifying the factors that contribute to variation in disease prevalence across host populations improves predictions of where parasites may have a strong impact and is critical for understanding how these relationships may shift with changing environmental conditions [10,11].

Despite the value of such information, for most host–parasite interactions we have little understanding of the relative importance of multiple host, abiotic and biotic factors on parasite prevalence [10,12]. Analysing geographical gradients and associated environmental variation offers a fruitful approach for disentangling the mechanisms underlying patterns in species abundances and interactions [13–15]. For instance, surveys and experiments along the Pacific coast of the USA have revealed that predation strength on intertidal mussels (*Mytilus californianus*) is influenced by a combination of water temperatures, predator traits and prey community composition [16–18]. This type of biogeographical approach has commonly been used to enhance our understanding of relationships among host and parasite diversity [19–21], but it has thus far been underutilized for understanding host–parasite interactions (but see [10,22,23]).

Understanding the ecological drivers of parasites is particularly important when they affect widespread or dominant host species that have cascading effects on community structure and ecosystem functioning [24–26]. For example, a yeast parasite in a freshwater crustacean host (*Daphnia magna*) resulted in a trophic cascade with ecosystem-level consequences. Specifically, parasite epidemics reduced host populations, which increased phytoplankton abundance and decreased water clarity [27]. Parasites can also affect species diversity; infection of the dominant marsh plant, *Salicornia virginica*, by the parasitic plant, *Cuscuta salina*, in a Californian salt marsh altered community composition and increased diversity by reducing the exclusion of inferior competitors [28,29]. Disease-induced mortalities of habitat-forming species may also reduce their three-dimensional structure and cause a decrease in the availability of shelter and resources for associated species [24,30].

The seagrass, *Zostera marina* (eelgrass), is an ecologically important foundation species that is ideal for understanding geographical variation in disease prevalence. *Z. marina* is a marine angiosperm that enhances local primary productivity, provides habitat for numerous invertebrates, fishes and birds, and contributes to nutrient cycling and sediment stabilization along coastlines throughout the Northern Hemisphere [31, 32]. In the 1930s, over 90% of *Z. marina* was lost in the North Atlantic as a result of seagrass ‘wasting disease’ caused by the parasitic protist *Labyrinthula zosterae* [33–35]. Recovery from these disease-induced losses has been inconsistent, and chronic infections continue to be reported [33,36–38]. Although the 1930s epizootic was centred in the North Atlantic, wasting disease infections have since been documented in seagrasses around the globe [39,40]. Further, some evidence suggests that aspects of ocean warming will increase the severity and prevalence of *L. zosterae* infections [41–43].

A number of host and environmental factors are known to influence the prevalence of wasting disease in eelgrass. For instance, lower salinities can inhibit wasting disease [44–46], higher nitrogen levels have been associated with increased wasting disease [47] and temperature exhibits a complex relationship with wasting disease depending on the context [41,43,44,48,49]. Additional research suggests that host traits such as eelgrass density, leaf length and genetic diversity also affect the prevalence of *L. zosterae* [38,42,50]. Yet, most of these studies were done in mesocosms, and field surveys to date have primarily focused on: (i) specific embayments [38,50]; (ii) mapping parasite distribution without measuring potential risk factors [37]; or (iii) a single key predictor such as temperature [43]. Thus, the relative importance of host and environmental factors in determining the large-scale distribution of wasting disease in the field remains an open question.

We quantified biogeographical patterns in wasting disease infection of eelgrass at 17 sites across 2 oceans in the Northern Hemisphere and tested for relationships with host and environmental factors. Specifically, we addressed the following questions: (i) How does wasting disease prevalence in eelgrass beds vary among oceans? (ii) What is the relative explanatory power of host traits (host size, density, genetic diversity and nutrient content), abiotic factors (seawater temperature, seawater salinity, latitude) and biotic factors (epibiont biomass, grazer abundance) for the prevalence of wasting disease in eelgrass beds? Given the documented differences in the distribution and characteristics of both host and parasite between the Pacific and Atlantic Oceans [33,51], we expected that wasting disease prevalence would vary between oceans. We also hypothesized that abiotic environmental factors, such as temperature and salinity, would be the strongest predictors of wasting disease distribution within oceans, both because they are strong predictors of disease distributions in other marine organisms [52–54] and because they are known to affect the parasite, *L. zosterae*, and host, eelgrass, that constitute the seagrass wasting disease pathosystem [45,46,55].

## 2. Material and methods

### (a) Study system

*Labyrinthula zosterae* is known to cause eelgrass mortality [36,56,57], yet spatial variation in prevalence is not well understood [36,37,40]. Physical contact with infected eelgrass tissue is the predominant mode of *L. zosterae* transmission among hosts [40], although waterborne transmission can also be important [58]. Following transmission, *L. zosterae* multiplies asexually within eelgrass leaf tissue [56]. Black and brown lesions resulting from leaf tissue necrosis are the most common signs of *L. zosterae* infection [56]. Necrosis caused by *L. zosterae* inhibits photosynthesis in infected tissue and can cause severe reductions in photosynthetic activity up to 5 mm away from lesions [59]. Once lesions have spread across the entire width of a leaf, photosynthetic activity of tissue towards the leaf tip is strongly inhibited, though the tissue may remain green [59]. Lesions also inhibit the vascular transport of nutrients, photosynthetic products or oxygen through leaf tissue [59].

### (b) Wasting disease survey

**Figure 1.**
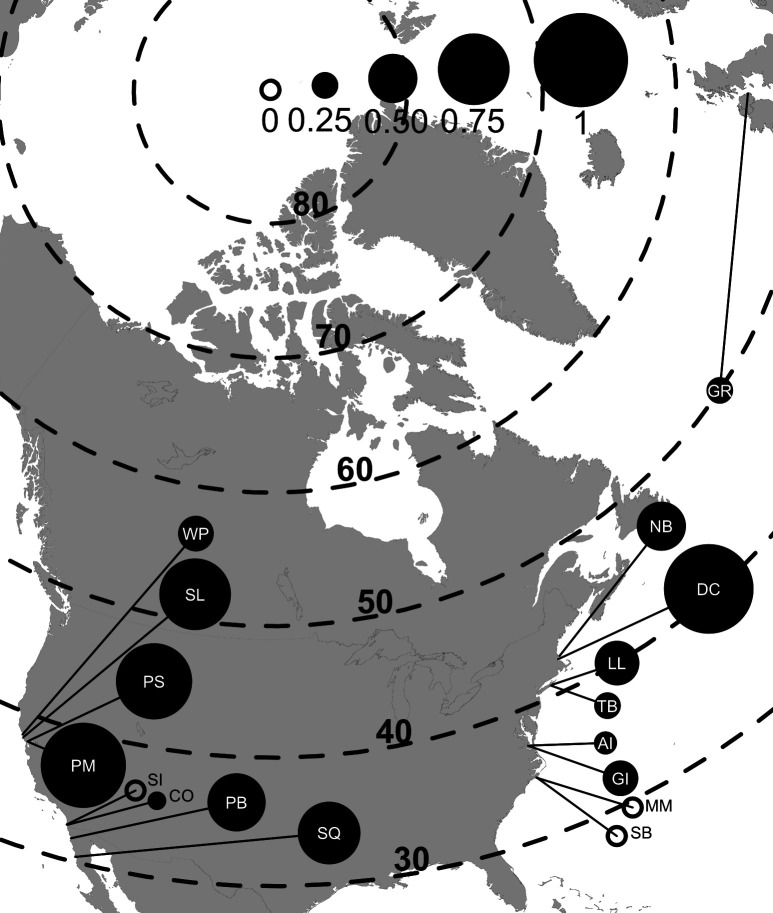
Map of study sites in the Northern Hemisphere. Wasting disease prevalence is indicated by the size of the circle associated with each location. Prevalence circles are labelled with two-digit site codes (see table 1). Dotted lines indicate latitude in increments of 10^o^.

In the summer of 2015, we surveyed 17 discrete, monospecific eelgrass beds across the Northern Hemisphere, mostly along the coasts of North America (nine in the Atlantic Ocean, eight in the Pacific Ocean; figure 1, table 1). All of the surveyed sites were in protected, shallow waters (0–2 m depth at low tide). At each site, we collected a total of 20 vegetative eelgrass shoots and scored the third youngest leaf of each shoot for lesions (electronic supplementary material, figure S1) [37]. We defined wasting disease prevalence as the proportion of the third youngest eelgrass leaves with lesions per site. We timed sampling to target peak wasting disease prevalence based on local knowledge of system dynamics, which resulted in most sites being surveyed in mid-summer near peak eelgrass biomass (table 1). Details of the lesion scoring method are described in electronic supplementary material, appendix S1.

**Table 1.** List of study site locations and dates *Z. marina* leaves were sampled and scored for wasting disease lesions.

site	site code	region	ocean	latitude	longitude	sampling date
Westside Park	WP	central California	Pacific	38.31976	−123.05514	16 Sep 2015
Sacramento Landing	SL	central California	Pacific	38.14964	−123.90638	3 Sep 2015
Point San Pablo	PS	central California	Pacific	37.97812	−122.40594	2 Aug 2015
Point Molate	PM	central California	Pacific	37.94656	−122.41850	1 Aug 2015
Shelter Island	SI	southern California	Pacific	32.71376	−117.22547	18 Aug 2015
Coronado	CO	southern California	Pacific	32.70076	−117.17289	18 Aug 2015
Punta Banda Estuary	PB	Mexico	Pacific	31.75847	−116.62278	20 Jul 2015
San Quintin Bay	SQ	Mexico	Pacific	30.41968	−115.96419	1 Aug 2015
Greyabbey	GR	Ireland	Atlantic	54.53056	−5.57056	18 Aug 2015
Niles Beach	NB	Massachusetts	Atlantic	42.59697	−70.65560	8 Aug 2015
Dorothy Cove	DC	Massachusetts	Atlantic	42.42014	−70.91544	5 Aug 2015
Landscape Lab	LL	Long Island Sound	Atlantic	40.85762	−72.45119	30 Jun 2015
Tiana Beach	TB	Long Island Sound	Atlantic	40.83158	−72.54082	30 Jun 2015
Allen’s Islands	AI	Virginia	Atlantic	37.25431	−76.43745	18 Jun 2015
Goodwin Islands	GI	Virginia	Atlantic	37.22042	−76.40134	3 Jun 2015
Middle Marsh	MM	North Carolina	Atlantic	34.69246	−76.62259	30 Jun 2015
Shackleford Banks	SB	North Carolina	Atlantic	34.67054	−76.57456	30 Jun 2015

### (c) Host and environmental surveys

In separate surveys at these same sites in 2014, we collected data on host and environmental factors that could mediate wasting disease prevalence. These data included eelgrass density (measured as shoots per m^2^), blade length (measured from the meristem to the tip of the longest leaf), leaf N content (from young leaf material of 5 pooled shoots in each plot run on a CHN analyser), allelic richness (calculated from 5 shoots per plot at 24 DNA microsatellite loci), periphyton (grams of total dried algae, microbes and detritus present on the leaf surface per gram of dried eelgrass) and mesograzer abundance (number of mesograzers per gram of eelgrass) following methods described in greater detail in Duffy *et al.* [15,51]. Many of these variables are generally consistent at a given site from year to year when sampled in the same season [60,61], and sometimes consistent across many years [62]. In addition, previous studies have demonstrated that variables measured months prior can be as or more predictive of wasting disease prevalence than those measured coincident with sampling of wasting disease [43,63]. However, we may be underestimating the predictive power of host and environmental factors on wasting disease by sampling them in consecutive years rather than the same year.

To quantify temperature and salinity at each site, we extracted estimates of the maximum mean monthly sea surface temperature and minimum mean monthly salinity from the Bio-ORACLE dataset for 2000−2014 (9.6 km^2^ resolution) [64,65]. We chose maximum temperature to capture summer conditions when past epidemics have been observed [57,66] and minimum salinity because lower salinities inhibit the parasite [45]. We used the raster package in R [67] to extract these data from all cells within 10 km of each site, and we averaged these estimates to generate site-level predictors. As a potential complement to these long-term data, we also measured *in situ* water temperature and salinity simultaneous with the wasting disease survey.

### (d) Analyses

In light of documented divergence in eelgrass form and genetics between the Atlantic and Pacific Oceans [51,68], we first used a generalized linear model with a binomial distribution to test for differences in wasting disease prevalence between oceans. Finding strong variation in prevalence between oceans, we then investigated the relationships between wasting disease prevalence and the suite of host (eelgrass shoot density, blade length, leaf N content and allelic richness), biotic (periphyton biomass and mesograzer abundance) and abiotic (latitude, *in situ* and long-term maximum sea surface temperature, *in situ* and long-term minimum salinity) factors in each ocean separately using random forests (RFs) [69]. We chose RF because it is a useful tool to extrapolate findings from local research across a larger area [70], and because it is robust both to collinearity among predictors and to the number of predictors included (i.e. overfitting [69–72]).

We used several approaches to validate our RF approach. First, recognizing that the strong collinearity in our dataset (electronic supplementary material, figures S2 nd S3) and the number of potential predictors relative to observations may push the limits of RF’s capabilities, we present the results of RFs on a reduced set of variables (*n* = 6: leaf blade length, shoot density, leaf N content, allelic richness, long-term maximum sea surface temperature, long-term minimum sea surface salinity) that omitted the predictors with high levels of collinearity and/or redundancy with other variables. RF models on the full set of predictor variables (*n* = 11) for each ocean produce results consistent with those from the reduced set (electronic supplementary material, figures S4–S6). In addition, we tested a range of different combinations of tuning parameters and found our results varied only slightly in terms of the absolute variable importance calculated from these different iterations and did not influence our interpretation of which variables were most important in either ocean. Details on RF methods and validation approaches are described in electronic supplementary material, appendix S2.

To gain ecological insight into the fitted RF models, we determined variable importance in terms of node impurity, which is computed as the per cent reduction in the residual sum of squares (RSS) that results from splitting the response variable based on each explanatory variable, and then averaging these values across all trees in the forest [71]. Explanatory variables that decreased node impurity most (greatest reduction in RSS) were interpreted to have greater importance for explaining variation in disease prevalence.

To visualize the relationship between each explanatory variable and response, we used partial dependency plots that show the average trend in the response variable (prevalence of wasting disease) as a function of the focal explanatory variable, while keeping all other explanatory variables in the model constant. Analyses were conducted using the randomForest package in R v. 3.6.1 [73].

## 3. Results

**Figure 2.**
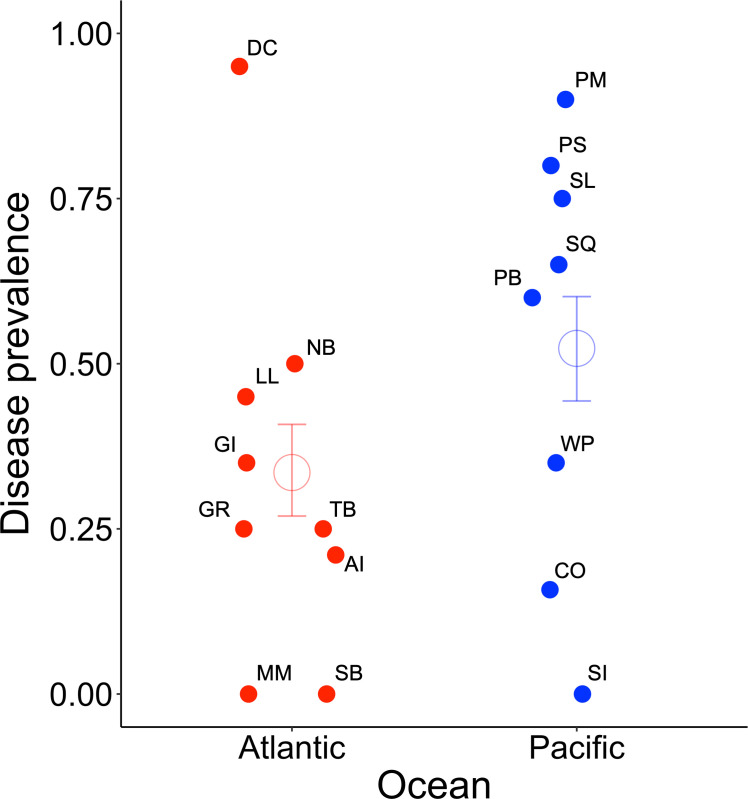
Wasting disease prevalence between oceans. The prevalence of wasting disease lesions was higher in the Pacific Ocean (blue symbols) compared with the Atlantic Ocean (red symbols). Error bars represent 95% confidence intervals around the mean (open points). Site prevalence is shown by filled points labelled with two-digit site codes (see table 1).

Wasting disease prevalence across sites in our survey ranged from 0 to >90% in both oceans (figure 2). However, prevalence in the Pacific (mean = 52.3%) was 1.5 times higher than in the Atlantic (mean = 33.5%; χ^2^_1_ = 11.813, *p* = 0.0006; figure 2). Our survey sites spanned 20° of latitude corresponding with *in situ* sea temperatures ranging from 10°C to 30°C, and *in situ* salinity values ranging from approximately 15 to 45 ppt. Consistent with the smaller latitudinal range of our samples in the Pacific Ocean (10°) versus the Atlantic Ocean (20°), the Atlantic sites spanned a greater range of environmental conditions (electronic supplementary material, figure S8). There was also considerable variation in host traits, with eelgrass densities ranging from fewer than 50 to over 1500 shoots m^−2^, and blade lengths from 10 to 100 cm (electronic supplementary material, figure S8). With the exception of blade length, which varied less across the sampled sites in the Atlantic than the Pacific, there was generally less variation between oceans in host versus environmental factors.

The RF model for the Atlantic Ocean with six predictors explained 12.69% of the variance in observed wasting disease prevalence, with a root mean square error of 0.11. The model for the Pacific Ocean explained 25.95% of the variance in the dataset, with a root mean square error of 0.12. There was also a strong relationship between observed and predicted wasting disease prevalence in both oceans (Atlantic: *R*^2^ = 0.95, *p* < 0.0001; Pacific: *R*^2^ = 0.95, *p* <0.0001; electronic supplementary material, figure S7). The models did exhibit some bias, with a tendency to predict more disease prevalence than was observed at the low end of the range and less prevalence than was observed at the high end of the range (electronic supplementary material, figure S7).

**Figure 3.**
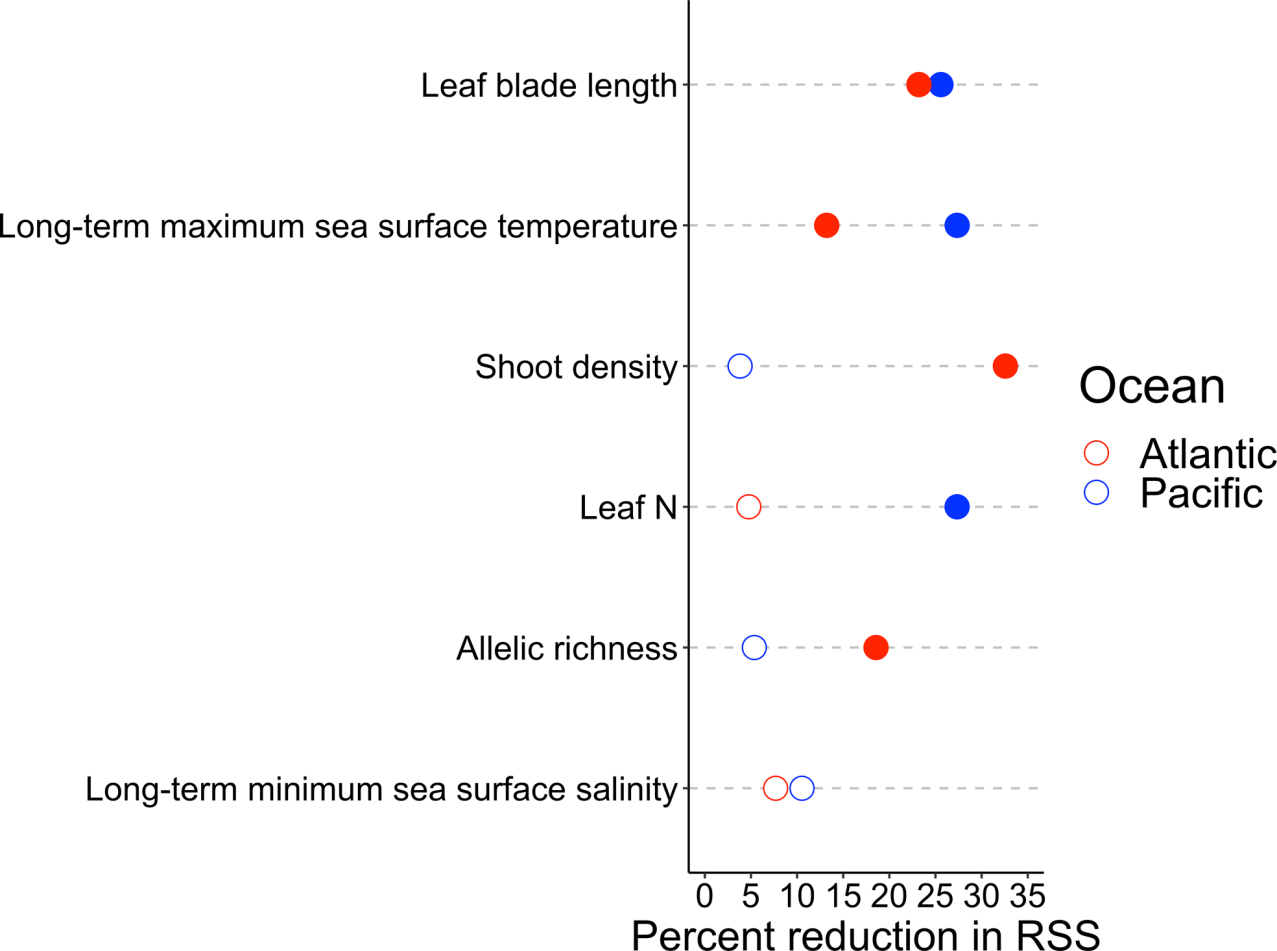
Relative importance of the wasting disease predictors in the random forest models (*n*_tree_ = 1000 and *m*_try_ = 3) for the Pacific Ocean (blue) and Atlantic Ocean (red). Filled circles indicate the subset of the most important variables that collectively explain at least 80% of the residual sums of squares (RSS), or the explanatory power of each variable, in each ocean.

The relative importance of the six predictors of wasting disease prevalence varied between oceans (figure 3). We focused on the variables that explained 80% of the overall reduction in RSS in the models; of the potential predictors, only long-term minimum salinity was not included in the top 80% for either ocean. In the Pacific Ocean model, three variables explained 80.3% of the reduction in RSS. Long-term maximum sea surface temperature, leaf blade length and leaf N each explained over 25% of the reduction in RSS (figure 3). Results from our analyses with the full suite (*n* = 11) of predictor variables were consistent with these three variables being the most important; among the remaining variables, long-term minimum sea surface salinity, periphyton biomass, *in situ* sea temperature and mesograzer abundance also contributed to 80% of the overall reduction in RSS, but none explained more than 10% of the reduction individually (electronic supplementary material, figure S4). In the Atlantic Ocean model, four variables explained 87.6% of the reduction in RSS. Here, host-related factors were the most important, with shoot density, blade length and allelic richness explaining approximately 33, 23 and 19% of the reduction in RSS, respectively, and long-term maximum sea surface temperature explaining approximately 13% (figure 3). Including the full suite of variables, periphyton biomass, *in situ* sea temperature and latitude, in that order, completed the list for the Atlantic (electronic supplementary material, figure S4). We summarize the form of these relationships below and note differences between oceans when appropriate.

**Figure 4.**
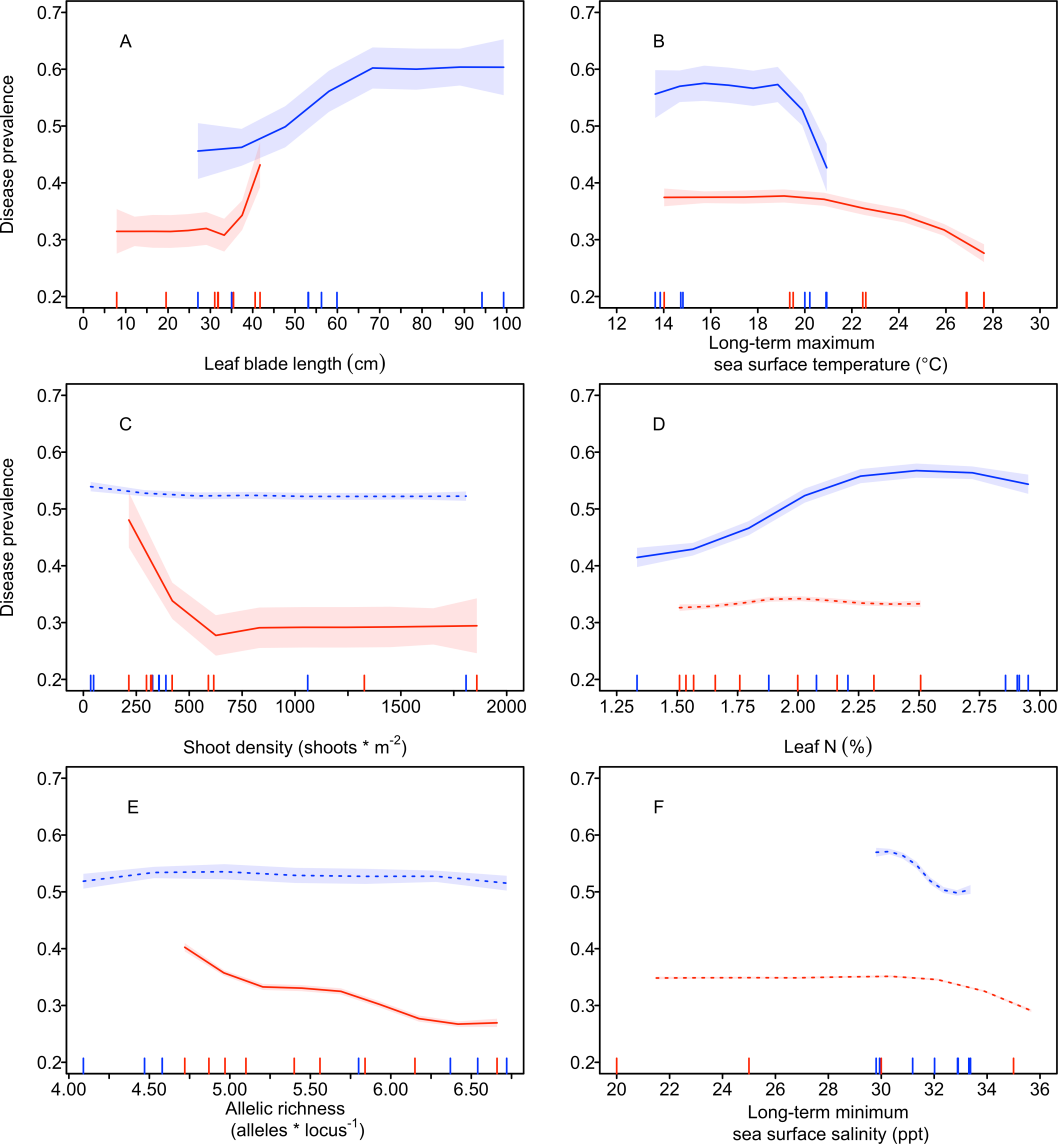
Partial dependency plots showing the trends in the prevalence of wasting disease lesions in the Pacific Ocean (blue) and Atlantic Ocean (red) as a function of each predictor, while keeping all other predictors constant. The curves were obtained via LOESS regression with 0.75 smoothing and the shaded regions represent 95% confidence bands. The notches on the *x*-axis represent the mean predictor values from each site. Factors are presented in the order of their mean residual sums of squares (RSS) across oceans, and filled circles and lines indicate the subset of the most important variables that collectively explain at least 80% of the RSS in each ocean.

Despite some variation in the relative importance of particular predictors, the nature of the relationship with wasting disease prevalence was generally similar between oceans for many of the key predictors. For example, wasting disease prevalence increased with increasing blade lengths, predominantly between blade lengths of 30−40 cm in the Atlantic and 35−65 cm in the Pacific (figure 4*a*). In addition, prevalence was lower at sites with warmer long-term maximum temperatures, with lower prevalence occurring at temperatures above approximately 20°C in the Pacific, with a more gradual decline observed above 20°C in the Atlantic (figure 4*b*).

Other predictors were related to wasting disease prevalence in one ocean, but not the other. In the Pacific only, wasting disease prevalence increased gradually with leaf N (figure 4*d*). In the Atlantic only, wasting disease prevalence was highest at sites with low shoot density (figure 4*c*) and decreased with increasing allelic richness (figure 4*e*). Relationships with the full suite of variables are shown in electronic supplementary material, figure S5.

## 4. Discussion

Our results reinforce the importance of both temperature and host characteristics (including morphology, condition and genetic diversity) for predicting variation in disease prevalence, although the shape of these relationships may vary. Wasting disease infection of *Z. marina* ranged from absent to nearly ubiquitous in sites distributed across >20° of latitude and two oceans, consistent with previous studies showing substantial variation in wasting disease at regional scales [37,38,41]. Although host characteristics explained most of the variance in eelgrass wasting disease prevalence in both the Pacific and Atlantic Oceans, the relative importance of those factors varied between oceans, adding to recent evidence for differences in eelgrass ecosystem structure between the Atlantic and Pacific, likely as a result of historical processes [51]. Long-term minimum salinity was not a significant predictor of wasting disease prevalence in either ocean, despite its significant effects on wasting disease severity in small-scale mesocosm experiments in both oceans (e.g. [45,46]). Overall, a comparison of our large-scale survey with prior region- and site-specific studies demonstrates that while some drivers of host–parasite interactions may scale up predictably, particularly those involved in basic transmission dynamics, the importance of others (e.g. salinity, leaf N, shoot density and allelic richness) depends on regional context and/or the range of values experienced.

Eelgrass wasting disease is spread by leaf-to-leaf contact and waterborne transmission, both of which can be facilitated by longer leaves and more densely packed shoots [36,38], and these transmission dynamics likely underpin the predictive value of host morphology in our study. For example, one study estimated that for every additional 1 cm of leaf length, the likelihood of a shoot having wasting disease increased by almost 2% [38]. This relationship may also contribute to the greater overall prevalence of wasting disease in the Pacific where eelgrass often grows in tall ‘forests’ compared with the Atlantic where eelgrass grows mostly in short ‘meadows’ [51]. The counterintuitive shape of the relationship between density and wasting disease prevalence in the Atlantic, with higher prevalence at lower densities, is primarily driven by the Dorothy Cove, MA site. This site, which exhibits a more ‘forest’-like structure with long leaves and lower densities than typical Atlantic ‘meadows’, appears to be the ‘exception that proves the rule’ regarding growth form differences between the Atlantic and Pacific and, thus, it is likely the longer blades are driving higher wasting disease at this site rather than lower densities. Alternatively, it could be that the direction of causality is reversed, and high wasting disease prevalence is driving low shoot densities due to mortality; however, we think this explanation is unlikely because this site has been regularly monitored for 10+ years with no observations of major die-offs or declines in density.

Temperature is a key determinant of disease dynamics [52,53,74], including for eelgrass wasting disease [41,43]. Many organisms, including parasites, exhibit unimodal thermal performance curves characterized by an initial increase in performance with increasing temperature up to a threshold, beyond which performance decreases rapidly. Temperatures exceeding these thresholds can limit the distribution of parasites in the field, especially those infecting ectothermic hosts [52,53,74,75]. Controlled infection experiments involving *L. zosterae* support a unimodal relationship, with increases in *L. zosterae* infection intensities between 11°C and 18°C and reductions between 22°C and 27°C in certain contexts [48,49]. Similarly, a *Labyrinthula* sp. isolated from another seagrass, *Posidonia oceanica*, exhibits a threshold temperature beyond which cell growth rapidly declines [76]. Consistent with these prior results, we found that the probability of wasting disease infection in both oceans decreased at sites where the maximum mean monthly sea temperatures reached above 20°C, with this threshold more pronounced in the Pacific than the Atlantic.

While our results suggest that warm waters (i.e. >20°C) may provide a refugium from wasting disease, several lines of evidence suggest that, in cooler climes, warming intensifies wasting disease infections for eelgrass. Specifically, recent studies in the eastern Pacific found that warm temperature anomalies are predictive of increases in disease prevalence and pathogenicity at sites with baseline temperatures around 9–12°C [41–43,63]. Smaller organisms such as parasites often acclimate more quickly to changing conditions than their hosts because of their higher mass-specific metabolic rates [77], and they may also evolve more quickly because of shorter generation times [72]. The resulting mismatches between host and parasite performance could underlie such increases in disease under warming [53,74]. In addition, for temperature-sensitive parasites that experience seasonal temperature variation, warming can increase the magnitude of peak prevalence and pathogenicity and extend the duration over which parasites are a problem [52,78]. In fact, the timing of our surveys coincided with the marine heatwave in the Pacific known as ‘the blob’, which was associated with an increase in eelgrass wasting disease [63] and other marine diseases (e.g. [79]). These anomalously high temperatures, not captured by our long-term maxima, could have contributed to the higher disease prevalence in the Pacific relative to the Atlantic observed in our study.

Finally, the pattern of decreasing disease prevalence with increasing temperatures that we detected does not exclude the potential for additional warming to increase wasting disease pathogenicity [37,46,66,80]. For example, independent responses of transmission and virulence of rickettsia-like parasites of black abalone to warming result in the decoupling of prevalence and pathogenicity [81]. Further refinement of the relationships among eelgrass, wasting disease and temperature is needed given the important implications for how this system will respond to continued warming.

Nutrients have been linked to elevated disease prevalence and severity in terrestrial, marine and freshwater systems, often due to changes in host quality [12,82,83]. For instance, a growth–defence trade-off, whereby plants with high tissue nutrient levels and high growth also have lower structural and chemical defences and increased disease, is a key driver of infection prevalence in terrestrial grasslands [83]. Consistent with this pattern, we found a positive relationship between leaf percent N and wasting disease prevalence in the Pacific Ocean (and no relationship in the Atlantic). Eelgrass growth is positively correlated with leaf length [84], which may contribute to the stronger nutrient and wasting disease relationship in the Pacific Ocean (where leaves are longer). Although we did not examine plant chemical defences, phenolics have been inferred as a defence mechanism of eelgrass owing to their increased concentration in diseased tissue [38,45], and prior work found higher nitrogen levels were associated with reduced production of phenolics and increased wasting disease [47]. While our leaf N results suggest the importance of direct effects of host nutrients on disease, effects of environmental nutrients could also influence disease prevalence via indirect mechanisms [83], as elevated water column nutrients have similarly been linked to increased wasting disease [85]. Despite prior evidence of reduced wasting disease prevalence at lower salinities [45,66,86,87], we did not detect that relationship here, perhaps because the salinities at our sites were above the threshold of 10−15 ppt where inhibitory salinity effects on *Labyrinthula* have generally been observed [45,87,88].

Wasting disease prevalence was lower in more genetically diverse eelgrass stands in the Atlantic (measured as allelic richness at DNA microsatellite loci), consistent with genetic diversity–disease relationships found in other systems [89–91], as well as a prior study in eelgrass [42]. Despite a similar range in allelic richness between ocean basins in the sites included in our study, this negative relationship was not present in the Pacific. When measured at the whole genome level rather than at the subset of microsatellite loci used in this study, eelgrass in the Pacific has much higher overall genetic variation than eelgrass in the Atlantic [68]. Therefore, sites in the Pacific may be over the threshold at which genetic diversity is important, in contrast to the less genomically diverse Atlantic where increases in allelic richness appear more important. The stronger effect of eelgrass genetic diversity on the disease when diversity is low is supported by the detection of eelgrass diversity–disease relationships in mesocosm experiments where diversity is also relatively low [42], and more broadly in diversity–function relationships [92]. Overall, the observed negative relationship between allelic richness and wasting disease prevalence adds to the considerable evidence for the ecological benefits of eelgrass genetic diversity [42,93–96] and highlights the importance of prioritizing the maintenance of diversity in conservation and restoration efforts, particularly in the Atlantic.

In our analyses including all possible predictors, community-level biotic characteristics, such as leaf periphyton and grazer abundance, were of similar importance to wasting disease prevalence as latitude and *in situ* abiotic variables, emphasizing the need to consider these characteristics in future examinations of wasting disease (see also [97]). For instance, the amount of periphyton on eelgrass leaves was a moderately important predictor of wasting disease in both the Pacific and the Atlantic Oceans. There was a much greater range of periphyton load in the Pacific than in the Atlantic, yet the relationship with disease prevalence remained mostly flat, with some indication of decreasing wasting disease prevalence as the load of periphyton increased in the Atlantic. These results are in contrast with prior findings that the odds of wasting disease increased considerably with greater epiphyte load [38]. Those authors speculated that epiphytes may cause a reduction in immune defences by blocking light and reducing photosynthesis, or through an indirect relationship with elevated nutrients that simultaneously increase epiphytes and decrease defences [38]. Alternatively, epiphytes may compete with the wasting disease pathogen for space on leaves and/or via interactions within and among the leaf microbiome, which may influence disease symptoms [42,98,99].

Differences in the relative importance and/or shape of the relationships between wasting disease prevalence and key factors between our large-scale survey and prior region- or site-specific studies highlight the importance of spatial variation in host and environmental characteristics, as well as the risks of over-generalizing results from site-specific surveys [23]. In addition, spatial variation in parasite traits, not examined here, could arise from coevolutionary interactions with their hosts and shape broad-scale patterns in disease (e.g. [100,101]). While phylogenetic analysis of *Labyrinthula* suggests only a single species, *L. zosterae*, causes wasting disease in eelgrass throughout the Atlantic and Pacific, isolates of this species from the two oceans vary in virulence [40,102]. Information regarding spatial variation in *L. zosterae* traits, and the factors driving this variation, is needed to advance our understanding of the seagrass wasting disease pathosystem, particularly given the increasing interest in assisted gene flow efforts that would move eelgrass, and potentially parasites, such as *L. zosterae*, over regional scales to mitigate climate-induced losses [103,104].

## Data Availability

Data are available at Dryad (https://doi.org/10.5061/dryad.5qfttdzh8) [105]. Supplementary material is available online [106].
